# Efficacy of immunoliposomes on cancer models in a cell-surface-antigen-density-dependent manner

**DOI:** 10.1038/sj.bjc.6601341

**Published:** 2003-10-14

**Authors:** S Hosokawa, T Tagawa, H Niki, Y Hirakawa, K Nohga, K Nagaike

**Affiliations:** 1 Pharmaceuticals Research Division, Mitsubishi Pharma Coporation, 1000, Kamoshida-cho, Aoba-ku, Yokohama 227-0033, Japan; 2 Department of Surgery, Kawasaki Municipal Hospital, Kanagawa, Japan; 3 Science and Technology Research Center, Mitsubishi Chemical Corporation, Yokohama, Japan; 4 Department of Physiology, Toho University, School of Medicine, Tokyo, Japan

**Keywords:** immunoliposomes, targeting, human monoclonal antibody, doxorubicin, polyethyleneglycol

## Abstract

We have recently established a cancer-reactive human monoclonal antibody, GAH, with a positive ratio of over 90% against stomach cancer. GAH was formulated as polyethyleneglycol (PEG)-modified immunoliposomal doxorubicin (DXR) (ILD) and its efficacy was examined against gastrointestinal human cancers. In *in vitro* studies, a comparison of ILD with PEG-modified liposomal DXR (LD) demonstrated that ILD had dose-dependent cytotoxicity for GAH-reactive B37 cancer cells, but not LD. In concordance with this result, microscopic observations showed that ILD was bound to and GAH-dependently internalised by B37 cells. In *in vivo* studies, ILD exhibited significantly greater antitumour activity on cancer xenograft models than LD or free DXR. The relation between efficacy and antigen density was examined on 10 xenograft models bearing cancer cells with varying GAH reactivity. Immunoliposomal doxorubicin therapeutic activity correlated with the antigen density, with a minimum number being required. Also, ILD revealed strong antitumour activity on cancers with low sensitivity to DXR or LD, suggesting that ILD overcame the DXR resistance of antigen-positive cancer cells. Thus, these results show that GAH endows liposomes with targeting activity, resulting in strong efficacy against gastrointestinal cancers.

Antibody-based targeting is a promising approach to the development of targeted cancer therapy (Bator and Reading, 1991), and liposomes are good candidates for drug delivery vehicles. Entrapping drugs in liposomes not only prolongs the drug circulation time but also changes the drug distribution *in vivo*. Moreover, significant progress has been made as a result of the development of liposomes modified with polyethyleneglycol (PEG), because they are taken up less by the reticuloendothelial system, thereby increasing their circulation time in the blood and their accumulation in tumours (Papahadjopoulos *et al*, 1991; Gabizon, 1992; Gabizon *et al*, 1994). Consequently, selective delivery of drugs to target cells can be achieved by liposomes tagged with antibodies that recognise specific determinants on the target cells (Ahmad *et al*, 1993; Park *et al*, 1995; Huwyler *et al*, 1997). Tumour-cell-specific antibody-conjugated immunoliposomes therefore can serve as drug vehicles with both liposome- and antibody-based targeting ability. We previously reported a specific therapeutic effect of anti-AFP and anti-CEA mouse monoclonal antibody (MAb)-conjugated liposomes containing doxorubicin (DXR) for an AFP-producing hepatoma (Konno *et al*, 1987) and a CEA-positive tumour (Uyama *et al*, 1994), respectively. These, and many other studies, have revealed the qualitative selectivity of immunoliposomes. Nevertheless, there has been no detailed report demonstrating a quantitative relationship between their therapeutic ability and the density of the antigens recognised by the conjugated antibody *in vivo*. We recently developed a human MAb (hu-MAb), GAH, which is highly reactive to human cancers, and in particular has an over 90% positive ratio against gastric cancer (Hosokawa *et al*, 2001). We report the construction of GAH-conjugated PEG-modified liposomal DXR (ILD) for cancer-targeting therapy and describe the cytotoxic activity of ILD and its targeting ability in a cell-surface-GAH-binding-site-density-dependent manner in both *in vitro* and *in vivo* studies against human xenografts.

## MATERIALS AND METHODS

### Chemicals

**Figure 1:**
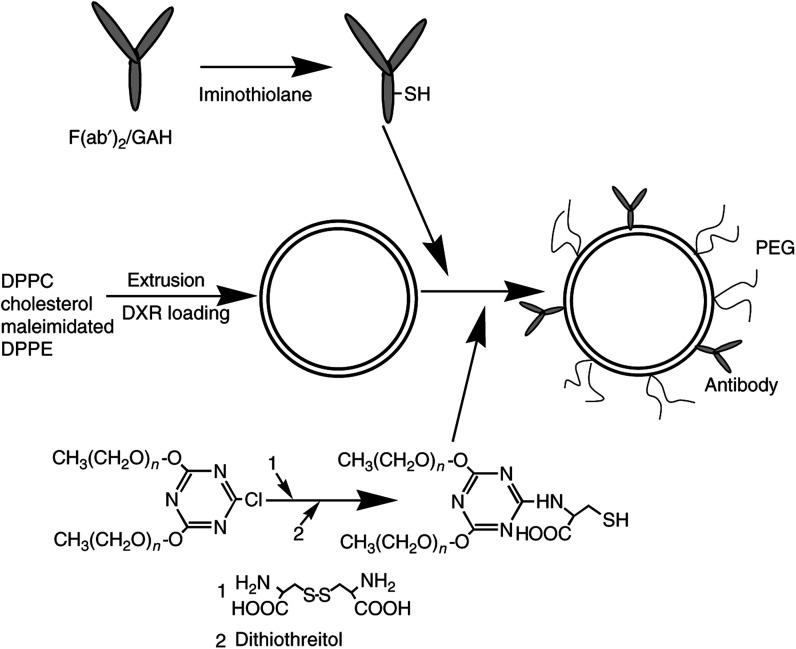
Preparation schemes for ILD and LD. Polyethyleneglycol and antibody were conjugated to liposomes through thioether linkage. Liposomal DXR was prepared in the same way as ILD, except for the antibody conjugation step. DPPC, dipalmitoylphosphatidylcholine; DPPE, dipalmitoylphosphatidylethanolamine.

Iminothiolane and 3(4,5-dimethylthiazoyl-2-yl)2,5 diphenyl tetrazolium bromide (MTT) were purchased from Sigma (Tokyo, Japan). 2,4,-*bis*-(PEG)-6-chloro-*s*-triazine, average PEG molecular weight of 5000, was obtained from Seikagaku Kogyo, KK (Tokyo, Japan). The two-chain-type PEG 2-[4,6-*bis*-(PEG)-1,3,5-triazin-2-ylamino]-3-mercapto-propionic acid was synthesised from L-cystine and 2,4,-*bis*-(PEG)-6-chloro-*s*-triazine (Figure 1

). A solid lipid mixture consisting of dipalmitoylphosphatidylcholine, cholesterol and maleimidated dipalmitoyl-phosphatidylethanolamine (18 : 10 : 0.5 molar ratio) were obtained from NOF Corporation (Tokyo, Japan). Doxorubicin hydrochloride was obtained from Kyowa Hakko Co., Ltd (Tokyo, Japan).

### Preparation of antibodies

GAH (IgG_1_, *κ*)-producing cells were established by the hybridoma technique (Hosokawa *et al*, 2001). In brief, lymphocytes obtained from a colon cancer patient were fused with P3U1 mouse myeloma cells to produce hybridomas. A hybridoma expressing an antibody, GAH, was selected based on cancer cell-specific reactivity against viable cancer cells by flow cytometric analysis. Reactivity was also confirmed by immunostaining of formalin-fixed cancer tissue sections. The results showed high rates of GAH reactivity to cells obtained from gastric cancer tissues and to gastric cancer tissue sections (>90%). The gene for GAH was cloned and expressed in Chinese hamster ovary cell to obtain a stable antibody-producing cell.

### Preparation of ILD and liposomal DXR (LD)

The F(ab′)_2_ fragments of GAH (F(ab′)_2_/GAH) were prepared by pepsin digestion of the intact GAH. The F(ab′)_2_/GAH was thiolated with iminothiolane (Traut *et al*, 1973). Immunoliposomal doxorubicin was prepared as depicted in Figure 1, essentially in the manner as described by Suzuki *et al* (1995) with some modifications. Briefly, the solid lipid mixture was hydrated to form multilamella liposomes and sized by passage through polycarbonate membrane filters (Nucleopore; Microscience) of sequentially smaller defined pore sizes, from 0.2 to 0.1 *μ*m. After DXR was loaded into the liposomes using the pH gradient method (Nichols and Deamer, 1976; Mayer *et al*, 1985), thiolated F(ab′)_2_/GAH and then PEG were conjugated with the liposomes through thioether linkage, respectively (Suzuki *et al*, 1995). Immunoliposomal doxorubicin was purified using Sepharose CL-6B to remove unincorporated DXR, unbound F(ab′)_2_/GAH and PEG. The incorporated amounts of the F(ab′)_2_/GAH and the PEG were determined by HPLC and these were 0.2 and 0.8 mg per 1 mg of DXR (10 mg in terms of total lipids), respectively. The final lipid concentration was typically 5–10 mg ml^−1^. The liposome size was measured with an ELS-800 dynamic light scattering instrument (Otsuka Electronics Co. Ltd, Osaka, Japan). The mean ILD size was 143 nm (ranging from 125 to 160 nm). Liposomal DXR was prepared in the same way as ILD, except for the antibody conjugation step. The incorporated amount of PEG was 0.8 mg per 1 mg of DXR (10 mg in terms of total lipids) and the mean size was 131 nm (ranging from 123 to 140 nm). Stability of the formulations was similar; it was found that the leakage from both formulations was less than 3% during 2 h incubation at 37°C or 2 weeks at 4°C in phosphate-buffered saline (PBS) (pH 7.4) determined by the method of Li *et al* (1998). The size change of both liposome formulations was less than 10 nm after the incubation periods.

### Cell lines

Human colon cancer cell lines Caco-2, DLD-1 and SW620 were obtained from the American Type Culture Collection, WiDr-Tc (human colon cancer cell line) and TE-8 (human oesophagus cancer cell line) were obtained from Tohoku University, Cell Resource Center (Miyagi, Japan). Human stomach cancer cell lines HSC-3, MKN-1 and MKN-45, and human rectum cancer cell line SW837 were obtained from IBL Co., Ltd (Gunma, Japan). B37 cells were established from a human stomach tumour (Hosokawa *et al*, 2001). Human venous endothelial cells (HUVECs) were obtained from Cell Systems Corporation (Kirkland, WA, USA).

### Animals

Male Balb/cAJcl-*nu* mice (4–5 weeks old) were obtained from Nihon Clea Co. (Tokyo, Japan) and were kept in standard housing. All animal studies were carried out according to the guidelines for the care and use of experimental animals, drawn up by the Committee for Animal Experimentation of Mitsubishi Pharma Corporation, which meet the ethical standards required by the law and the guidelines about experimental animals in Japan.

### *In vitro* comparison of the cytotoxic activity of ILD and LD

B37 cells were seeded on a 96-well plate (Costar) at 5 × 10^3^ well^−1^. After 1 day of culture, the cells were incubated for 1 h at 37°C with ILD or LD diluted with normal human serum to a DXR concentration of 0–10 *μ*g ml^−1^. The cells were then washed with medium and cultured for 6 days with 10% FCS-containing medium. Viable cells were detected by MTT assay (Green *et al*, 1984).

### GAH-reactivity-dependent cytotoxic activity of ILD

B37 cells and HUVECs were cultured to confluence. Immunoliposomal doxorubicin or free DXR was added to the cells at a DXR concentration of 1 *μ*g ml^−1^, and the cells were cultured for 2 more days. The cells were examined through a microscope for viability.

### Internalisation of ILD by cancer cells

ILD was labelled with PKH2 (Zynaxis Cell Science, Inc., Malvern, PA, USA), which emits green fluorescence and has a high affinity for the lipid bilayers generally used for membrane labelling (Horan *et al*, 1990). Immunoliposomal doxorubicin was incubated with PKH2 labelling solution for 10 min at 60°C. Unbound PKH2 was removed with a NAP-10 column. Cultured B37 cells were reacted with 20 *μ*g ml^−1^ of labelled ILD, according to the DXR concentration, for 1 h at 4°C without or with free F(ab′)_2_/GAH at a concentration of 0.5 mg ml^−1^. For an additional control, the cells were incubated together with free human IgG F(ab′)_2_. After another 1 h incubation at 37°C, the cells were washed and fixed with 2% paraformaldehyde. Then the cells were examined for the distribution of green fluorescence under a confocal fluorescence microscope (LSM-510, Carl Zeiss) equipped with an argon laser.

### *In vivo* therapeutic studies

#### Subcutaneous (SC) model

Male Balb/cAJcl-*nu* mice were inoculated subcutaneously on the back with one million MKN-45 cells. When the tumour xenografts were fully established (11 days after implantation), mice were assigned to different treatment groups. Liposomal DXR or ILD was administered intravenously (i.v.) three times at weekly intervals at a DXR equivalent dose of 2.2 mg kg^−1^. Free F(ab′)_2_/GAH (0.36 mg kg^−1^) mixed with LD was injected using the same schedule. The control group was treated with saline. The tumour size was measured with a caliper and the weight was calculated as ½ × length × width^2^ (Geran *et al*, 1972). The ratio of the estimated tumour weights compared to those of the first day was determined and compared for each group.

#### Subrenal capsule (SRC) tumour model

Male Balb/cAJcl-*nu* mice were implanted under the renal capsule (Bennett *et al*, 1985) with a few mm^3^ pieces of cancer xenografts of HSC-3, B37, WiDr-Tc, SW837, TE-8, Caco-2, DLD-1, SW620, MKN-1 and MKN-45, which had been prepared from SC passages. The mice were divided into four groups, and treatment was started the next day (day 1). The experimental groups of mice received three i.v. injections, 3.0 mg kg^−1^, of free DXR, ILD or LD, respectively, at 6-day intervals (on days 1, 8 and 15). The control group was treated with saline. The tumours were excised and weighed on day 22.

### Statistical method

Differences in *in vivo* antitumour activity were analysed by Dunnett's two-tailed test. All statistical analyses were conducted with an SAS System statistical program, version 6.12 (SAS Institute, Inc., NC, USA).

### Quantitative measurement of cancer cell surface antigens

Viable cells were isolated from tumour tissues transplanted to nude mice. In brief, fresh tumour tissue was minced with a razor blade and suspended in medium. The suspension was passed through a nylon mesh and centrifuged to remove the necrotic debris. The separated cells were allowed to react with FITC-labelled GAH at a concentration of 50 *μ*g ml^−1^ on ice for 60 min. These cells were washed and resuspended in PBS containing propidium iodide (PI). FITC and PI fluorescence intensities per cell were measured with a flow cytometer (FCM) (FACS 440 or BD-LSR; Becton/Dickinson). Dead cells with PI fluorescence due to incorporation of PI into their nuclei were excluded from the data. The number of GAH molecules bound to the viable cell surface was calculated using fluorescent latexes with known FITC-bound molecule numbers (Flow Cytometry Standard) as standards.

## RESULTS

### Role of GAH conjugation in liposome-mediated cytotoxic activity of ILD on cancer cells

**Figure 2:**
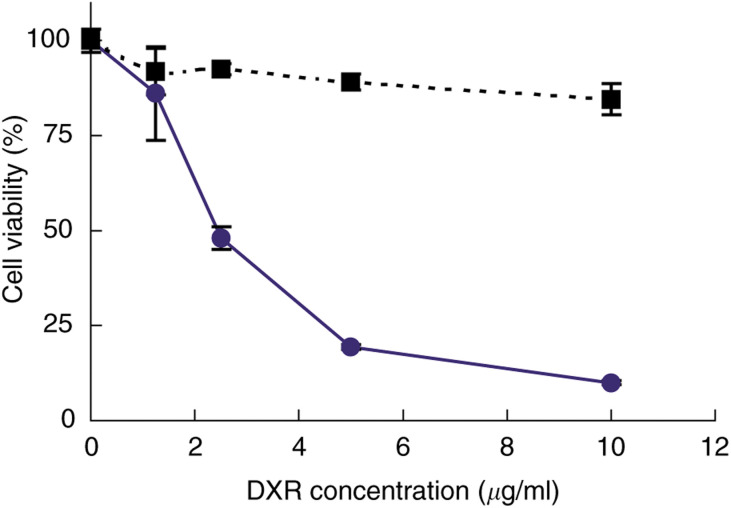
Comparison of the antiproliferative activity of ILD and LD against B37 cells. Cells in a 96-well plate were exposed to ILD (•) or LD (▪) at the doses indicated, and the relative cell growth was determined by MTT assay. Each value is the mean±s.d. of four determinations except at the DXR concentration of 1.25 *μ*g ml^−1^ (*n*=3).

First, to confirm the function of GAH conjugation to liposomes, the activities of ILD and LD against GAH-reactive B37 cells were compared using MTT assay. The ILD showed strong dose-dependent cytotoxicity for B37 cells, despite the short-term treatment, whereas the LD had no significant toxicity in the dose range used (Figure 2

). These results indicate that ILD exerts its cytotoxic activity through GAH conjugated to the liposome surface.

### Internalisation of ILD by cancer cells

**Figure 3:**
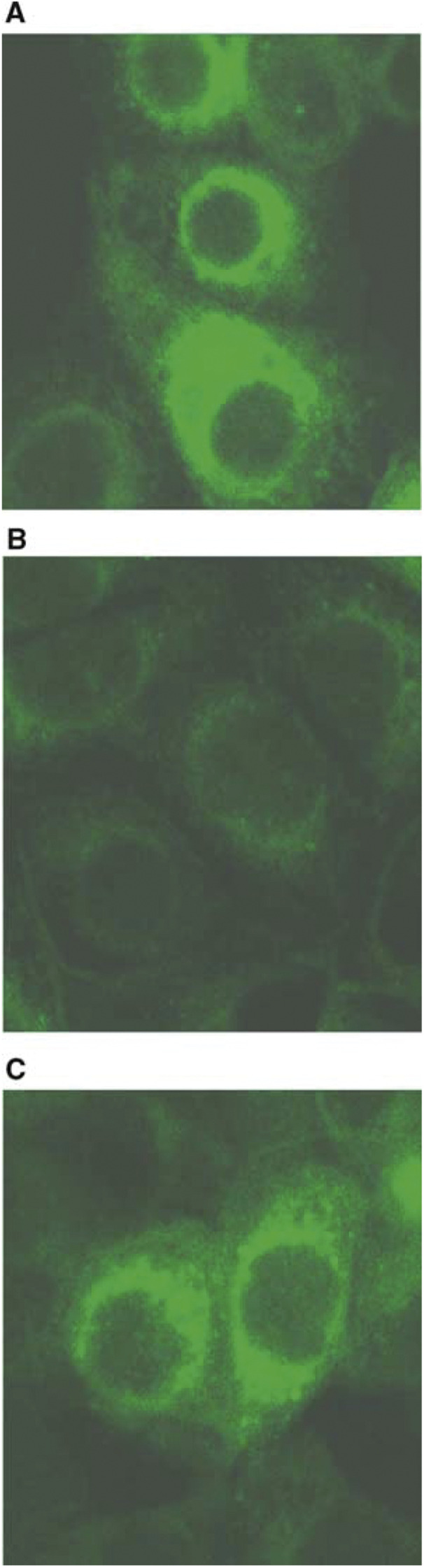
Internalisation of ILD. Cultured B37 cells were reacted with PKH2-labelled ILD for 1 h at 4°C without (**A**) or with free F(ab′)_2_/GAH at a concentration of 0.5 mg ml^−1^ (**B**), about 100 times the amount of antibody bound on liposomes, as well as with free human IgG F(ab′)_2_ (**C**) for a control. After another 1 h incubation at 37°C, the cells were washed and fixed and then examined under a confocal fluorescence microscope. Scale bar: 10 *μ*m.

In order to identify the mechanism of the reaction between ILD and cancer cells, cultured B37 cells were incubated with PKH2-labelled ILD. The fluorescence distribution was then examined. Confocal microscopy visualised an accumulation of intracellular fluorescence in the cells incubated with ILD (Figure 3A

). This intracellular accumulation was inhibited by the addition of free GAH (Figure 3B), whereas hardly any inhibition was observed in a control experiment using human IgG (Figure 3C). This showed that the cancer cells internalised the ILD and that the internalisation was mediated by GAH binding to the cell surface.

### Comparative efficacy of ILD on HUVEC and cancer cells

**Figure 4:**
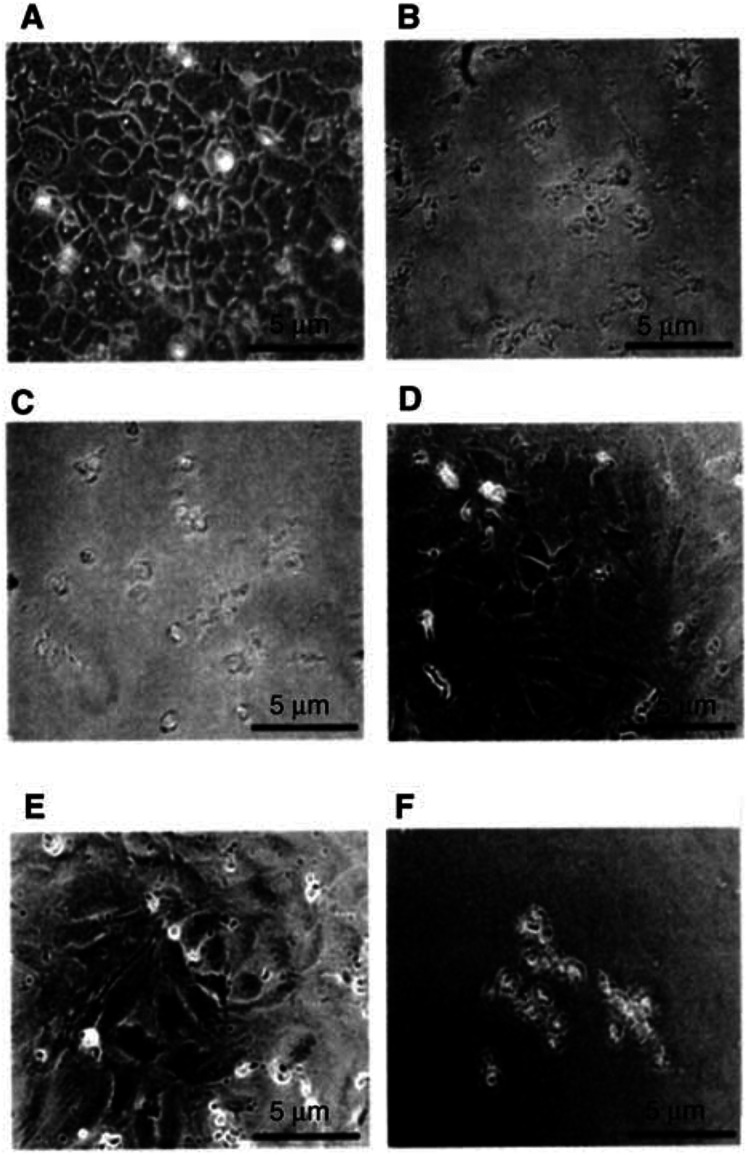
GAH-reactivity-dependent cytotoxicity of immunoliposomes. B37 cells (**A–C**) or HUVECs (**D–F**) were cultured for 2 days with ILD (**B, E**) or free DXR (**C, F**) at 1 *μ*g ml^−1^ in terms of DXR concentration. (**A**) and (**D**) show drug-free controls for B37 and HUVECs, respectively. Cell viability was assessed microscopically. Scale bar: 5 *μ*m.

GAH is known to be unreactive to normal tissues and cells including HUVECs. To determine the tumour *vs* normal efficacy of ILD, cell viabilities of B37 and HUVECs were compared after 2 days of culture with ILD or free DXR at a concentration of 1 *μ*g ml^−1^, which is a convincing concentration range based on clinical DXR dosage. Free DXR was cytotoxic against both B37 cells and HUVECs (Figure 3C and F). On the contrary, ILD showed no significant cytotoxicity on GAH-non-reactive HUVECs (Figure 4E

), comparable to a drug-free control (Figure 4D), while the cytotoxicity of ILD against B37 cells was remarkable (Figure 4B). These findings indicate that ILD has GAH-reactivity-dependent cytotoxicity.

### Efficacy of ILD against antigen-positive gastric cancer evaluated with xenograft models

**Figure 5:**
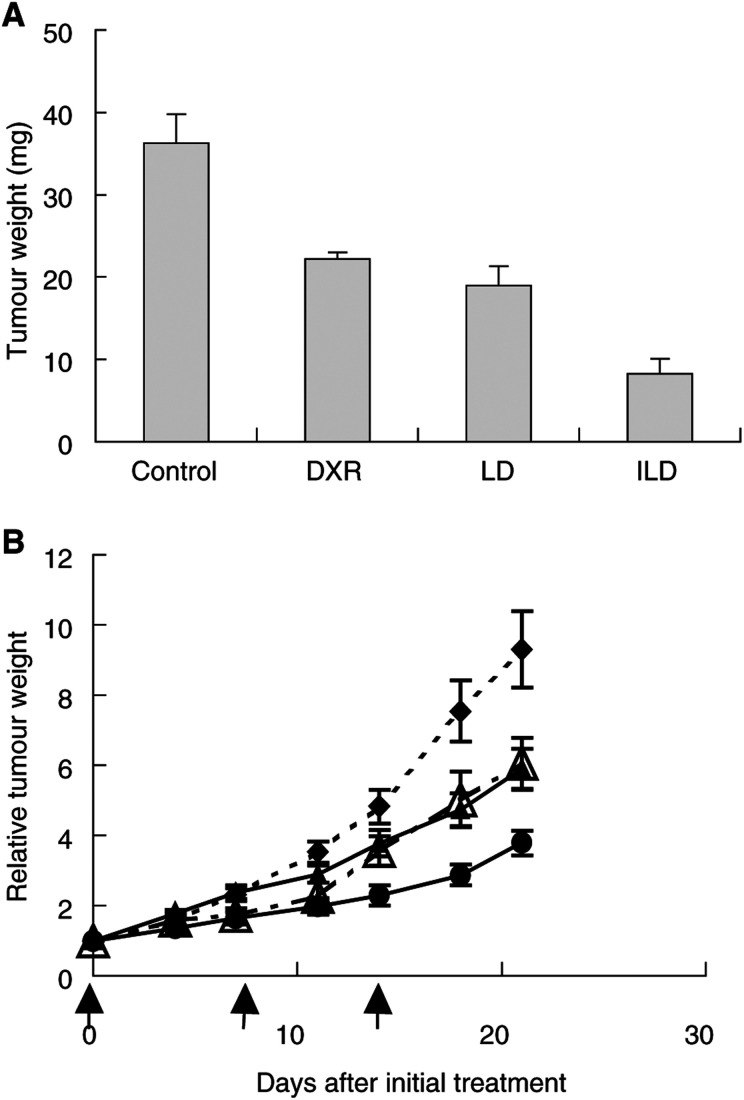
Comparison of therapeutic effects of ILD, LD and free DXR. On SRC models (**A**), ILD, LD or free DXR was injected three times i.v., with 3.0 mg kg^−1^ in terms of DXR, into nude mice (*n*=6) bearing MKN-45. The antitumour effect of ILD was significantly higher than that of LD (*P*<0.05) or DXR (*P*<0.01). Bars: means± s.e. MKN45 cells implanted in SC (**B**) were treated with LD (▴, *n*=13), ILD (•, *n*=12), free F(ab′)_2_/GAH (0.36 mg kg^−1^) mixed with LD (▵, *n*=6), at a DXR equivalent dose of 2.2 mg kg^−1^, or saline (♦, *n*=12), by i.v. injections on the 3 days indicated (↑). Change in tumour weight was determined over the treatment period. The antitumour effect of ILD was significantly higher than that of LD (*P*<0.05) or LD supplemented with free GAH (*P*<0.05). Bars: mean±s.e.

The therapeutic effect of ILD was examined on MKN-45 human gastric cancer xenograft models because MKN-45 has been used for *in vivo* evaluation of immunoliposomes (Uyama *et al*, 1994) with excellent reproducibility, and it has a high GAH antigen expression level. The antitumour efficacy of ILD was compared with those of free DXR or LD with an MKN-45 SRC xenograft model. The result showed that ILD activity was significantly greater than that of DXR or LD (Figure 5A

). When ILD activity was compared with that of LD on an MKN-45 SC model (Figure 5B), significantly higher antitumour activity of ILD was confirmed in concordance with the SRC result. Furthermore, LD mixed with free GAH (0.36 mg kg^−1^) was administered for an additional comparison. As a result, supplementation with free GAH gave no distinguishable difference in LD antitumour activity, indicating that the ILD antitumour effect resulted from the conjugation of the antibody onto liposomes, and not synergistic activities of LD and antibody, nor the result of modulation of drug sensitivity caused by antibodies.

### Estimation of number of bound GAH per viable cancer cell

To analyse the relation between the antitumour activity of these formulations and the number of antigens, the number of bound GAH molecules per viable cancer cell (designated as Ag density) was determined by the FCM analysis described above. The average numbers for each cancer cell line were as follows: Caco-2, 3.9 × 10^4^ cell^−1^; DLD-1, 5.5 × 10^4^ cell^−1^; SW620, 7.4 × 10^4^ cell^−1^; WiDr-Tc, 3.7 × 10^5^ cell^−1^; SW837, 9.9 × 10^4^ cell^−1^; TE-8, 2.0 × 10^5^ cell^−1^; HSC-3, 7.0 × 10^4^ cell^−1^; MKN-1, 1.0 × 10^5^ cell^−1^; MKN-45, 1.2 × 10^5^ cell^−1^; and B-37, 1.1 × 10^5^ cell^−1^.

### Quantitative evaluation of the effect of antigen density on ILD activity in the *in vivo* model

**Figure 6:**
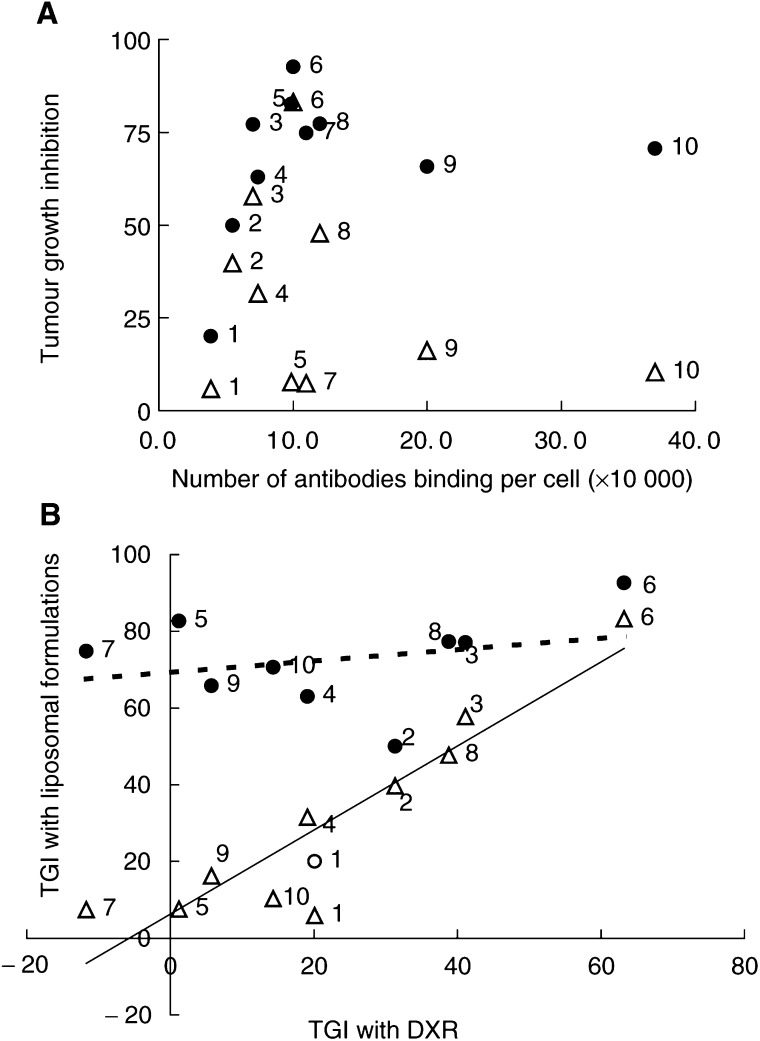
Quantitative GAH-binding-site-density-dependent effect of ILD. SRC models were established with 10 cancer cell lines having different numbers of GAH binding sites, as noted in the experimental section. These nude mice were treated with drugs in the same manner as described in Figure 5. From the results of each therapeutic experiment, tumour TGI observed with ILD or LD was plotted against the number of GAH-binding sites (**A**) or against DXR sensitivities (**B**), which were expressed as tumour TGIs obtained with free DXR. Open triangles, LD; closed circles, ILD against antigen-positive cells. In **B**, the open circle is a result of ILD against Caco-2 cells, which were regarded as a antigen-negative control. Numbers next to symbols show the data points from the following cancer cells: 1, Caco-2; 2, DLD-1; 3, HSC-3; 4, SW620; 5, SW837; 6, MKN-1; 7, B-37; 8, MKN-45; 9, TE-8 and 10, WiDr-Tc.

The relation between antitumour activity and Ag density was examined using 10 human cancer cell lines on SRC models. Tumour weights, as a result of treatment with ILD, LD or DXR in each cancer model, were obtained in the same manner as for MKN-45 SRC xenograft. From these values, tumour growth inhibition (TGI) for each drug was calculated as follows: (1−(average tumour weight of a drug treatment group)/(average tumour weight of a control group)) × 100. Figure 6A

depicts TGIs for ILD or LD against Ag density on each cancer. A correlation was found between Ag density and TGI treated with ILD (Figure 6A, closed circle), while no statistical relation between TGI with LD and Ag density was observed (Figure 6A, open triangle). In particular, ILD showed antitumour activity superior to that of LD against cancer cells when Ag density was >1 × 10^5^ cell^−1^, whereas the superiority of ILD over LD was not observed on Caco-2 (cell line numbered 1 in Figure 6), for which the number was as low as 3.9 × 10^4^ cell^−1^. Liposomal DXR showed lower efficacy against many of the cell lines tested, but excellent efficacy against a few of them (Figure 6A). Hence, the activities of both liposomal formulations were further analysed in terms of DXR sensitivity on each cancer cell tested *in vivo*; TGI treated with DXR was used as an index for this sensitivity as shown in Figure 6B. The results showed that LD antitumour activity was likely to be reflected in DXR sensitivity, unlike ILD (Figure 6B). In other words, LD activity might be limited by DXR sensitivity of cancer cells, while ILD would be likely to surpass the limit and able to overcome the drug resistance of those cells when they have enough Ag density. Against DLD-1 (numbered 2 in Figure 6) or Caco-2, ILD showed modest TGIs, which were plotted among the data points of LD activity. It was thought that the minimum Ag density required for this immunoliposome to overcome the DXR resistance of cancer cells was more than the density of Caco-2 cells (Figure 6B, open circle).

## DISCUSSION

In order to utilise the newly established human antibody GAH fully, the antibody was formulated as an immunoliposome system where F(ab′)_2_/GAH, with Fc portion removed, and PEG were incorporated into the surface of liposomes containing DXR. This novel immunoliposome was evaluated *in vitro* and *in vivo* against gastrointestinal cancers.

GAH dependency on the character of ILD was revealed by the selective cytotoxicity against B37 cells as well as the internalisation by these cells in the *in vitro* studies (Figures 2 and 3). Also, ILD did not show nonspecific cytotoxicity to the antigen-negative HUVECs (Figure 4). Although the endothelial uptake of liposomes is reported (Papadimitriou and Antimisiaris, 2000; Laverman *et al*, 2001), this formulation was found to be substantially inert with the normal cells, but active with the target cells.

In the *in vivo* study, enhanced ILD activity was shown with both the conventional SC model and the SRC model, on which it has been demonstrated that many human tumours grow well and retain the morphology and characteristics of the parent tumours (Aamdal *et al*, 1985). Liposomal DXR showed a modest, but better antitumour effect compared with free DXR in all gastric cancer xenograft models tested, which could be attributable to enhanced liposomal accumulation in tumour tissue (Papahadjopoulos *et al*, 1991), known as passive targeting. However, it is also known that liposomes localise in the tumour extracellular compartment and are not taken up by tumour cells (Gabizon, 1992; Huang *et al*, 1992), suggesting that the LD acts as a local depot of DXR.

On this point, Park *et al* (1997) and we (Maruyama *et al*, 1997) revealed distribution of liposomes with tumour xenograft models, showing that antibody conjugation on liposomes does not necessarily lead to increased tumour accumulation over plain liposomes. It is postulated that the accumulation of (immuno)liposomes was governed by passive diffusion and extravasation from tumour blood vessels, and that improved antitumour activity of immunoliposomes over plain liposomes can be explained by the differences in intratumoral localisation or internalisation (Park *et al*, 1997). In fact, when evaluated using a B37 xenograft model, GAH immunoliposomes led to the accumulation of a similar amount of DXR in tumour mass as LD did. In order to clarify the role of GAH antibody for immunoliposomal targeting against solid tumour, we examined the efficacy of ILD against tumour cell lines having a variety of antigen densities.

We found ILD activity superior against stomach and colorectal cancers *in vivo*; the activity of ILD correlated well with cell-surface-GAH-binding site density (Figure 6A). Together with the fact that ILD internalised against target cells (Figure 3), superior therapeutic activity of ILD was thought to result from passive targeting as well as active targeting, the effect of GAH conjugation. These results also suggest that in order to exert an immunoliposomal targeting effect fully, a minimum Ag density might be required; it seemed that the threshold lay between 1 × 10^4^ and 1 × 10^5^ cell^−1^, determined by the method mentioned herein. In concordance with these observations, Kirpotin *et al* (1997) showed antigen-density-dependent targeting of anti-Her2 immunoliposomes *in vitro*. More recently, Park *et al* (2002) demonstrated that anti-Her2 immunoliposomes exhibit excellent antitumour efficacy *in vivo* against Her2-overexpressing breast tumour cells, while the efficacy was almost the same as that of nonimmunoliposomes against low-Her2-expressing MCF-7 cells (1 × 10^4^ receptor cell^−1^). In view of DXR sensitivity, our results suggested that ILD could overcome DXR resistance of cancer cells *in vivo* (Figure 6B). We actually observed that ILD possesses therapeutic efficacy against DXR-insensitive stomach cancer cells, which express a multidrug resistance gene (data not shown). This property would have special importance in treating gastrointestinal cancers clinically, as they often show drug resistance against many types of anticancer drugs. The evidence that ILD was internalised by the target cells might serve as one avenue for enabling immunoliposomes to overcome the drug resistance, as demonstrated by Suzuki *et al* (1997) that an immunoliposome targeting transferrin receptor (internalising receptor) modulates DXR resistance in DXR-resistant human leukaemia cells.

GAH-conjugated immunoliposome characteristics are thought to depend on GAH characteristics. It has been suggested that GAH recognises a molecule related to cytoskeletal components present on the surfaces of cancer cells. Investigators have recently shown that modified cytokeratin molecules (Ditzel *et al*, 1997) or vimentin-like protein (Nasoff *et al*, 1997) is present on the surface of some malignant cells, and they are recognised by hu-MAbs produced by cancer patients' lymphocytes. The extent and nature of these unique antigens are being investigated, and they are expected to become targets of immunoconjugates for cancer therapy as shown here.

We showed that GAH endows liposomes with targeting activity, and allows for their internalisation by cancer cells. These properties could result in strong efficacy and might be able to overcome the drug resistance of cancer cells. Together with its high positive ratio against stomach cancer, we believe that this immunoliposome could be a versatile drug-targeting tool for the treatment of gastrointestinal cancers.
